# NURSING PRACTICE AND OPTIMAL DELIRIUM CARE AMONG OLDER ADULTS IN ACUTE CARE SETTINGS

**DOI:** 10.1093/geroni/igae098.3354

**Published:** 2024-12-31

**Authors:** Tanya Mailhot, Zineb Bouaouina, Tania Valentine Joannette, Imene Khetir, Patrick Lavoie

**Affiliations:** Université de Montréal, Montreal, Quebec, Canada; Université de Montréal, Montreal, Quebec, Canada; Université de Montréal, Montreal, Quebec, Canada; Montreal Heart Institute, Montréal, Quebec, Canada; Université de Montréal, Montreal, Quebec, Canada

## Abstract

Studies have emphasized the limited availability of data regarding the current practices of nurses in delirium care for patients in acute care settings. Similarly, there is a lack of information regarding the obstacles and facilitators in providing optimal care for the prevention, detection, and management of delirium.

**Aim:**

To describe nurses’ practices about delirium care in acute care patients and their perceptions about barriers and facilitators regarding optimal care. Method: A two phase, multi method design was used. The quantitative phase utilized a self-reported survey to assess nurses’ knowledge, practice, confidence, and collaboration regarding delirium care. The qualitative phase employed focus groups to complement and explore survey data in-depth. Recruitment took place on nine acute surgical and medical units across two university-affiliated hospitals in Canada, involving nurses in direct patient care.

**Results:**

231 nurses reveal diverse insights into delirium care in acute settings and survey participants showed solid knowledge of delirium symptoms and outcomes, yet 23% did not identify the hypoactive form’s prevalence. While 81% reported receiving information on delirium screening, challenges included time constraints and misuse of detection tools. Qualitative themes highlighted communication’s vital role, challenges posed by delirium presentations, the significance of staff support, time constraints, the impact of experienced staff, the role of families, and the importance of additional resources for optimal delirium care.

**Conclusion:**

Findings align with existing literature, emphasizing the multifaceted nature of delirium care and the need for tailored approaches, education, and collaborative strategies to improve overall care quality.

